# 11- to 13-Year-Old Children’s Rejection and Acceptance of Unfamiliar Food: The Role of Food Play and Animalness

**DOI:** 10.3390/nu15061326

**Published:** 2023-03-08

**Authors:** Rikke Højer, Margit Dall Aaslyng

**Affiliations:** Nutrition and Health, Campus Slagelse, University College Absalon, Sdr. Stationsvej 30, 4200 Slagelse, Denmark

**Keywords:** children, tactility, animalness, health promotion, hands-on strategy, food literacy, food behavior, food acceptance, food rejection, food categorization

## Abstract

Promoting children’s healthy food behavior is important in reducing the risk of developing obesity; it is therefore relevant to investigate methods to promote healthy food choices. This study’s aim was to investigate differences in rejection–acceptance mechanisms related to unfamiliar foods depending on the inclusion of tactile exercises prior to cooking and food origin. Participant observation was applied in a school setting. Eight fifth and sixth grade classes were recruited from four Danish schools (*n* = 129). The classes were divided into two groups: animal (AG; quail) and nonanimal (NAG; bladderwrack). AG and NAG were subdivided into two groups: food print (FP) and no food print (NFP). Applied thematic analysis was applied. During preparation/cooking, NFP displayed disgust-related rejection, whereas FP displayed inappropriateness-related rejection. FP exhibited more playful behavior. Inappropriateness and animalness drove AG rejection. NAG rejection was driven by the slimy texture of the food and the perception of it ‘not being food’. Acceptance was driven by taste and familiarity. In conclusion, the inclusion of tactile exercises could increase children’s exploratory food behavior, and the promotion of children’s healthy food behavior should not solely focus on choosing foods deemed safe and familiar, since, despite rejection during cooking, acceptance is ultimately possible.

## 1. Introduction

### 1.1. Background

Promoting healthy food behavior from childhood is an important target area. In 2016, 18% of the world’s children and adolescents aged between 5 and 19 years were either overweight or obese [1]. In Denmark, 18% of 9- to 13-year-old children are overweight [2]. Childhood obesity is associated with an increased risk of developing, for example, low self-esteem, type 2 diabetes (T2D), and cardiovascular disease (CVD) [3,4].

Breastfeeding during infancy and exposure to a variety of new foods during the complementary feeding period has been shown to moderately reduce the risk of childhood obesity and promote self-regulation of intake and preference for healthy foods later in life [1,5]. As children become older and more independent, the family arena is partly replaced with the school arena together with influence from peers on food behavior. The World Health Organization [1,6] recommends that schools implement programs that promote healthy school environments, health, nutrition, food literacy, and physical activity among school-age children and adolescents in order to reduce childhood obesity and promote life-long healthy food behavior.

The school is a relevant arena for the promotion of culinary skills since a survey of Danish children’s participation in home cooking activities has shown that children’s participation in cooking the family evening meal decreased from 2020 to 2022. In 2020, 68% of Danish children participated in the cooking of the evening meal once a week. In 2022, only 58% participated [7,8]. Furthermore, the school arena is relevant since previous studies have found that children who participate in cooking activities have an increased enjoyment of cooking in later life, a greater willingness to taste novel foods, an increase in self-esteem regarding the choice of healthy foods, and increased preference for healthy food [9,10,11,12,13,14,15].

Culinary skills are embedded in the concept of food literacy, which Vidgen and Gallegos [16] define as follows: ‘[…] *a collection of inter-related knowledge*, *skills and behaviours required to plan*, *manage*, *select*, *prepare and eat food to meet needs and determine intake. This can simply be interpreted as the tools needed for a healthy lifelong relationship with food*’ [16] (p. 54). The concept of food literacy can be applied to the promotion of healthy food behavior since it includes functional (knowledge), interactive (skills), and critical components (transformation and empowerment). Food literacy is not only associated with knowledge and skills related to cooking. It is also about promoting a person’s ability to make critical and reflective health and food choices (for example, knowing what foods to eat and why, how to read food label information, etc.) whilst recognizing that individual, social, cultural, and environmental experiences with food affect one’s ability to navigate the food system [16,17,18]. According to Pendergast, Garvis, and Kanasa [19], the ability to navigate the complex food system can be achieved through the development and promotion of self-efficacy, which is a belief in one’s ability to cope with and take required action when engaging in a given task and/or situation [20,21]. The concept of food literacy has been applied as a framework in prior health promotion interventions [22,23,24].

According to Rozin and Fallon [25,26], the taxonomy of food acceptance and rejection is driven by three main motivations: sensory-affective factors (e.g., liking/disliking taste or smell), anticipated consequences (e.g., negative/positive physiological or social), and ideational factors (e.g., knowledge of the nature or origin of the food). Motivations for rejecting food are distaste (all sensory perceptions, real or imagined [27,28]), danger, inappropriateness, and disgust. Motivations for accepting food are good taste, beneficial, appropriate, and transvalued [27,29]. Rozin and colleagues’ [25,29,30] taxonomy of rejection and acceptance has been applied in a variety of food behavior studies (e.g., [24,27,28,31,32,33]). Furthermore, Sick, Højer, and Olsen [32] found that dislike of taste and appearance and bad smell were among the most common reasons for children rejecting food, whereas curiosity was a driver of acceptance.

Højer, Wistoft, and Frøst ([33], Figure 8, p. 12) suggested a rejection–acceptance continuum, which illustrates the movement between rejection and acceptance based on how children categorize food (exemplified by fish: from first exposure to the fresh fish until the fish has become a meal through cooking). The continuum categories were animal, nonanimal, animal, and food, where the categories ‘animal’ promoted rejection (animal 1: seeing the whole fresh fish, touching it, washing it; animal 2: filleting the fish), whereas the categories ‘non-animal’ (gyotaku exercise/fish printing) and ‘food’ (cooking and eating) promoted acceptance. They concluded that tactile play could be a relevant tool in promoting children’s acceptance of food. In previous studies, tactility or tactile play have also been suggested as drivers in promoting acceptance of healthy food [34,35,36,37].

This study focuses on children’s acceptance and rejection of unfamiliar food in order to shed light on tools that could be used in promoting children’s healthy food behavior with particular reference to official Danish dietary guidelines, for example, eating a varied diet and eating less meat from four-legged animals (max. 350 g of meat in total per week) [38].

### 1.2. Aim of the Study

The aim of this study was to investigate if there is a difference in the rejection–acceptance continuum mechanisms in relation to unfamiliar food items for children aged 11 to 13 years depending on (1) whether or not a tactile exercise is included prior to cooking and (2) whether the food is of animal or nonanimal origin.

## 2. Materials and Methods

### 2.1. Study Design

**Figure 1:**
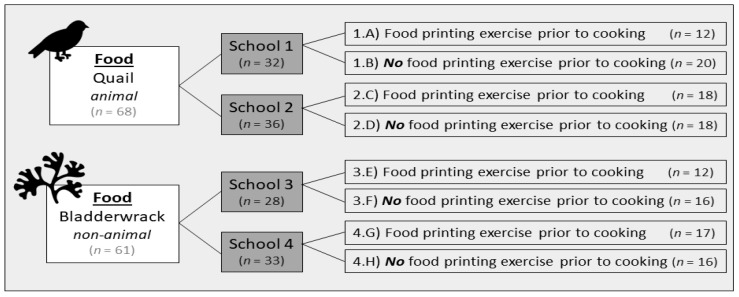
Study Design.

The study design was an intervention with multiple cases [39]. Eight different classes (fifth and sixth grade) from four different schools were included in the study, as shown in Figure 1.

Two schools each had two classes working with a food item categorized as ‘animal’: quail (Coturnix coturnix), and two schools each had two classes working with a food item categorized as ‘non-animal’: bladderwrack (*Fucus vesiculosus* Linnaeus (1753)). At each school, one class completed a food printing exercise prior to cooking, whereas the other class did not.

### 2.2. Participants

Eight classes from fifth and sixth grades (11 to 13 years of age) were recruited from four different Danish public schools (*n* = 129; boys: *n* = 57; girls: *n* = 72). The schools were situated in the region of Zealand. The recruitment was conducted through an existing network by sending out information letters via e-mail to schools in the eastern part of Denmark addressed to the schools’ food knowledge teachers. Before the study started, each participant’s legal guardian provided written informed consent. The participating children were also asked to provide written informed consent even though this was not legally necessary. This was done due to ethical considerations related to the inclusion of the participating children as recommended by the Danish National Council for Children [40].

### 2.3. Setting and Materials

#### 2.3.1. Setting

The intervention took place in a natural setting at the children’s schools in the school teaching kitchen as part of the subject of food knowledge. Classes carried out the exercises based on the same food-specific exercise guide. Six trained research assistants conducted the exercises in two teams. Each team conducted all the exercises within the same category (animal/nonanimal). In each case, the class teacher was present during the exercise.

At the school, the children were already divided into four kitchen groups, and therefore, since the exercise took place as part of a formal subject, the existing groups were not altered. The exercise was carried out over two consecutive lessons (2 × 45 min.).

#### 2.3.2. Materials

Four exercise manuals (two for animal and two for nonanimal: quail or bladderwrack; food print/no food print) were developed and tested internally by the research team prior to the intervention to ensure feasibility of recipes, level of difficulty, time frame, and uniformity in communication.

The food printing exercises for both animal and nonanimal categories were modified from the traditional Japanese fish printing technique Gyotaku [41]. The following materials were used: squid ink diluted with tap water in a cup, a small sponge, five A4 pieces of paper cut into eight equal parts, paper towels, and printing paper (Chinese rice paper).

**Figure 2:**
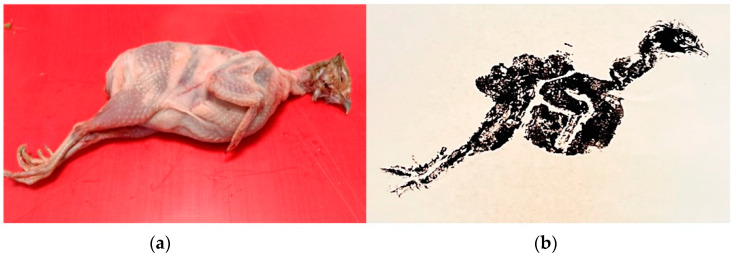
(**a**) Fresh European quail (Coturnix coturnix); (**b**) food print of fresh European quail.

The animal category subject was European quail (Coturnix coturnix) from breeding stock (see Figure 2a). In both exercises (food print/no food print) in the animal category, the children had to wash the quail and debone it in preparation for a dish: roasted quail stuffed with apples, cinnamon, and butter. The children who did the food printing exercise prior to deboning and cooking would start by washing the quail, drying it with paper towels, placing cut paper squares around and slightly under the quail (to avoid getting excess squid ink on the print later in the process), and covering the quail with squid ink using the sponge. Then, the paper squares were removed, and the squid ink-covered quail was covered with the printing paper. Using gentle strokes, the squid ink was transferred to the paper, and when the paper was lifted, a mirror print of the quail would appear (see Figure 2b). All children in the food printing groups were given the opportunity to create their own print.

**Figure 3:**
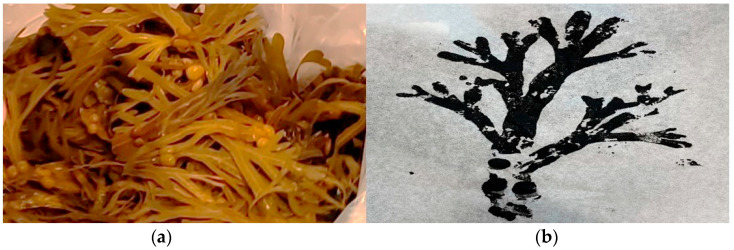
(**a**) Fresh bladderwrack (*Fucus vesiculosus*); (**b**) food print of fresh bladderwrack.

The nonanimal category subject was bladderwrack (*Fucus vesiculosus*) Linnaeus (1753), which is an edible brown macroalga (Ochrophyta, Phaeophyceae) (seaweed) [42] (see Figure 3a). In both exercises (food print/no food print) in the nonanimal category, the children had to wash the bladderwrack in preparation for a dish: pasta with bladderwrack pesto and bladderwrack chips. The children who did the food printing exercise prior to cooking would follow the same printing exercise procedure as in the animal category (see Figure 3b for result).

### 2.4. Data Collection

The following qualitative data collection methods were applied in this study: participant observation [43] and situational photography as a supporting method [44].

A loosely constructed participant observation guide was drawn up, allowing observations to follow the rejection–acceptance continuum categorizations [33] and the phases in the exercise (see Table 1).

Furthermore, the participant observation guide was developed based on the taxonomy of food rejection and acceptance [25,29]. It was primarily concept-driven [45], but the structure of the guide also left room for exploratory inquiry.

Documentation methods used during the participant observation comprised written field notes and situational photos to document various situations and child–food interactions. The field note strategy was inscription and transcription [45]: Descriptions of behaviors (inscriptions) and informants’ own words and dialogues (transcriptions) were documented in an observational journal.

If the children asked what had been written in the journal, they were given the opportunity to read it and comment on it. Furthermore, if children refused to touch, handle, and/or taste the bladderwrack or quail, this was respected by the researchers. Data were collected in October 2021.

### 2.5. Data Analysis

Data were analyzed using applied thematic analysis (ATA) developed by Guest, MacQueen, and Namey [46]. Thematic analysis has been applied as a data analytical method in previous food behavior studies (e.g., [47,48,49]).

**Figure 4:**
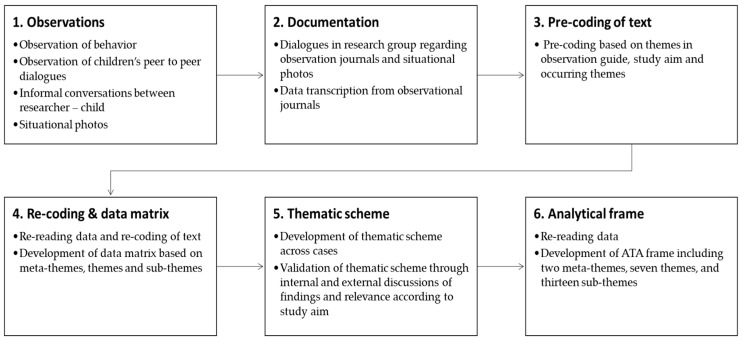
Overview of ATA data processing.

The concept-driven [45] data processing (see Figure 4 for an overview of the ATA process) was based on four metathemes related to the exercise flow and model frame of the acceptance–rejection continuum [33]: animal/seaweed, nonanimal/not seaweed, animal/seaweed-food, and food. Precoded text was organized in a matrix based on the frequency of observed behaviors and conversations related to rejection and acceptance.

**Figure 5:**
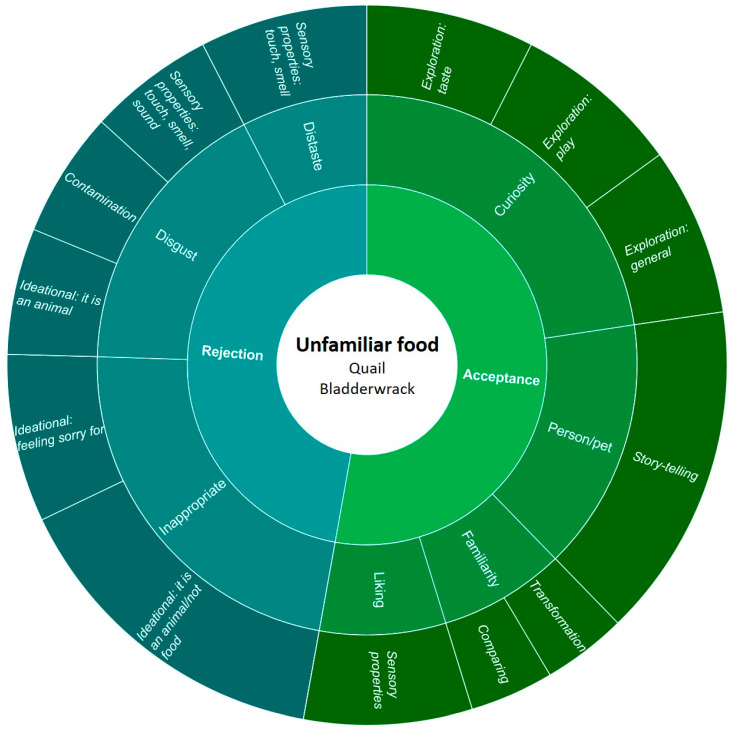
ATA frame overview: Metathemes, themes, and subthemes across cases.

A thematic scheme was developed to investigate possible themes and subthemes across cases (see Appendix A Table A1). A rereading of the data set and a reconsideration of subthemes and themes were performed to ensure accurate representation and relevance in accordance with the study aim [46,50]. The ATA process resulted in two metathemes, rejection and acceptance, and seven themes, inappropriate, disgust, distaste, curiosity, person/pet, familiarity, and liking. Furthermore, thirteen subthemes were identified (see Figure 5 for a presentation of the ATA frame).

Data without relevance to the study aim were excluded from the analysis. Furthermore, the ATA frame (analysis, results, and discussion thereof) was discussed within the research group (the essence of metathemes and themes is available in Table A2).

## 3. Results

The data are presented according to the ATA frame (Figure 5) and study aim. The following abbreviations are applied in the presentation of the results: QP = quail—food print group; QNP = quail—no food print group; BP = bladderwrack—food print group; BNP = bladderwrack—no food print group; AG = animal-origin group (QP + QNP); and NAG = non-animal-origin group (BP + BNP).

### 3.1. Inclusion of Tactile Exercise: Impact on Rejection and Acceptance Continuum Mechanisms

#### 3.1.1. Metatheme Rejection: Disgust, Distaste, and Inappropriateness

Thematically, no differences between the QP and QNP were observed in relation to rejection and the categorization of the quail as an animal throughout the experiment. Nevertheless, differences were observed in how the children handled the quail during the preparation phase (animal categorization according to the acceptance–rejection continuum).

Children who had not engaged in the food printing exercise displayed their rejection through disgust related to a contamination dimension to a greater extent than the children who had participated in the printing exercise. The latter was more driven by inappropriateness in the form of feeling sorry for the quail:

QNP: Before starting the deboning, they have to remove the remaining feathers from the quail. Several only touch the quail with their fingertips or only with tweezers. Many of the children do not want to touch the quail with their other hand (to hold the quail steady on the cutting board) (School 2D, 21).
QP: As they are about to cut off the quail’s head, a girl holds her hands over her quail’s eyes so it will not see it (School 1A, 60).

Similarly, no thematic differences were observed between the BP group and BNP group in relation to rejection and the categorization of the bladderwrack as a nonanimal/raw food throughout the experiment. Nevertheless, differences were observed in how the children handled the bladderwrack during the preparation phase (resembling the animal categorization according to the acceptance–rejection continuum).

The BNP group displayed a higher degree of disgust based on the contamination factor during the preparation and cooking phase compared to the BP group. For example, children in the BNP group were very concerned about getting too close to the bladderwrack and would carry it between two fingertips, holding it away from the body. The BNP group also displayed a more pronounced distaste, particularly with regard to smell:

BNP: A boy says to a girl: ‘just f****** touch it’. The girl replies: ‘*it smells*’ (School 3F, 100).

In addition, the subtheme inappropriateness related to the ideational theme “it is not food” was evident in the BNP group. It was particularly related to the bladders on the bladderwrack. Several groups chose to remove them before making pesto or chips. This did not seem to be an issue in the BP group.

#### 3.1.2. Metatheme Acceptance: Curiosity, Person/Pet, Familiarity, Liking

Even though no differences in acceptance and categorization between the QP and QNP were observed throughout the experiment, differences were observed in how the children reacted in the (pre-) preparation phase and the meal/tasting phase.

The QP displayed a higher degree of food play after the printing. They would touch the quail all over and, for example, make it fly and make up small stories about the quail. This was also observed in the QNP, though to a lesser extent.

Children who had participated in the food printing exercise were highly driven by curiosity related to the exploration of the taste of the quail dish, and references to familiar food were made (e.g., ‘*tastes just like chicken*’, ‘*best cutlet ever*’). However, children from the QNP tasted the quail but were more concerned with the animal dilemma:

QNP: ‘*If you didn*’*t know what you were eating*, *you would eat it quickly*’. Response: ‘*WE ARE EATING!*’ (said in an accusing manner) (School 2D, 59).

Furthermore, observations indicated that the QP was generally faster and better at deboning the quail than the QNP.

Thematically, no differences between the BP and BNP groups were observed in relation to acceptance and categorization of the bladderwrack throughout the experiment. Nevertheless, differences were observed in how the children in the two groups reacted in the meal/tasting phase (food categorization according to the acceptance–rejection continuum).

In the BP group, the children displayed a more curious exploratory approach to tasting the dishes with bladderwrack than the BNP group:

BP: Tasting the pesto: A girl tastes the pesto while another girl reminds her that it has seaweed in it. The girl who is tasting covers her mouth with her hand as if in surprise and replies: ‘*the aftertaste is actually very good*’ (School 4G, 184–187).

In the BNP group, there seemed to be concern related to gathering the courage to taste. For example, one child in the group would taste before the rest of the group.

### 3.2. Animal—Nonanimal Origin: Impact on Rejection and Acceptance Continuum Mechanisms

#### 3.2.1. Metatheme Rejection: Inappropriateness, Disgust, and Distaste

Both the AG and NAG categorized their food item as inappropriate, though the way in which the item was inappropriate differed between the two groups. Inappropriateness in the AG was based on two approaches to the ideational dimension: ‘feeling sorry for’ and ‘it’s an animal’, whereas the NAG focused ideationally on the bladderwrack not being food.

Feeling sorry for the quail in the AG was particularly displayed in the preparation phase during deboning:

AG: ‘I really feel sorry for it’ (School 1B, 20).
AG: ‘If you cut off its head, you are an animal abuser’ (School 2D, 4).

Furthermore, observations also found that feeling sorry for the quail was closely related to the perception that, even though the quail was dead before they got it, they were the ones killing it during the deboning phase.

In the AG, the element of the inappropriateness of it being an animal was present in the introduction, picking up, and deboning phases. During the introduction and picking-up phases, the inappropriate animalness was related to visual cues, and some children said that they could not kill a small chicken or that they did not slaughter animals, but just ate them. During the deboning phase, the dimension of killing drove the perception of animalness together with the knowledge that the quail was an animal and/or had had a life:

AG: A girl looks at the quail before starting to debone: ‘*YUCK! It is a little bird*’ (School 1A, 71).

In the NAG, inappropriateness was linked to bladderwrack being perceived as not being food, especially during the preparation phase. The bladders in particular were regarded as not being food, and they were removed. In addition, before the bladderwrack was added to the pesto mix and blended in a food processor and during the blending, it was also not regarded as food:

NAG: Preparation of pesto: Group 2 begins to blend their pesto. They scream when the bladderwrack makes noises: ‘*YUCK!*’, ‘*what is this?*’ (School 4H, 76).

Furthermore, a display of mixed inappropriateness (not food) and disgust and a fear of contamination was observed in the NAG since the group did not want to mix their pesto with the pasta even though this was the serving style recommended in the recipe.

Both the AG and NAG displayed disgust in handling their food item, particularly during initial physical contact, picking up the food item, and the preparation phase. The reactions of disgust displayed in both groups were characterized as a fear of contamination driven by the tactile properties of the food items. The bladderwrack was slightly slimy, which resulted in excessive rinsing, with some groups rinsing several times followed by a thorough drying of every single bladderwrack. In addition, the bladders led to contamination-related observations, with the children arguing about the content of the bladders (e.g., puss, pimples, “do not want to know”). In the case of the quail, a fear of contamination was particularly linked to coming into contact with waste products:

AG: ‘I do not want dead bird on me’ (School 2C, 47).

During the preparation phase, disgust was displayed through reactions related to sensory properties. In the AG, particularly, the sound of cutting through the skin and bones of the quail resulted in a display of disgust, but also touch, closely related to contamination, and smell were factors that promoted reactions of disgust (e.g., turning away, mimicking vomiting). Disgust-related sensory properties in the NAG were displayed as reactions to smell (by mimicking vomiting) and touch, closely related to contamination, which was displayed in the way the bladderwrack was handled (e.g., carrying it away from the body, holding it with only two fingertips, not touching during rinsing but turning the bowl around to move the bladderwrack).

Rejection related to distaste sensory properties were displayed in both the AG and NAG. However, only visual and touch-related cues were present in the AG, though the NAG also displayed distaste related to smell and touch.

For the AG, distaste related to either visual cues or touch was primarily connected to inappropriateness in the introduction, picking up, and preparation phases. For example, during the introduction phase, the children said that they were not going to eat THAT (the quail), and during the preparation phase, touching the quail was avoided by not holding it while cutting (even though they had had no problem touching it when printing).

In the NAG, distaste was also displayed as touch avoidance (but without outbursts and mimicking vomiting), and references to bad smell were made, particularly during the preparation of chips and pesto:

NAG: Group opens the oven and agree that it is smelly (from the bladderwrack chips). They really do not like the smell (School 3F, 73).

The NAG displayed distaste in the meal/tasting phase related to the taste of the food, which was not the case for AG. In particular, the taste of the chips was not liked, which resulted in several children spitting them out after tasting them.

#### 3.2.2. Metatheme Acceptance: Curiosity, Pet/Person, Familiarity, Liking

Both the AG and NAG displayed curiosity when handling their food item. For example, general exploration was displayed in the AG at the beginning of the preparation phase before deboning. The children explored the quail’s beak by opening it up and checking for teeth, lifting the wings, etc., and, during the deboning process, they explored whether the quail contained an egg (several did). In the NAG, general exploration was primarily done after initial contact (after rinsing) and as part of the beginning of preparation. In particular, the children would explore by smelling the bladderwrack after rinsing, and they were observed to be speculating on what the bladders were. Exploration through food play was also observed in both the AG and NAG, though food play was much more pronounced in the AG than in the NAG. In the AG, examples of food play included lifting the quail up and pretending that it was flying (preparation phase, before deboning), playing with the viscera, and playing with the carcass (preparation phase, during/after deboning). Food play in the NAG was isolated to try to poke holes in the bladders during preparation. Curiosity related to exploring taste was primarily related to the meal/tasting phase in both the AG and NAG, but the way in which the exploration of taste was handled differed between the two groups. In the NAG, there was a more cautious approach since the pesto was not mixed with the pasta but served separately, so if the pesto (an unfamiliar ingredient in a familiar food) was not liked, it was still possible to eat the pasta (known food). In the AG, the animal dilemma was a factor, with several children commenting that it had been a live animal.

The theme of personification/petification was observed to be present only in the AG, and it was very much displayed during food printing and during deboning in the preparation phase. The children would make up small stories about their quails and give them names:

AG: During food printing: ‘This is Jens, and this is Birgitte. They are married’ (School 1A, 31).
AG: During the deboning, a girl finds an egg in her quail. She takes the head of the quail, which has been cut off earlier, and shows the egg to the head, saying: ‘congratulations, you have become a father!’ (School 1A, 83).

Familiarity was observed to be a theme related to the promotion of acceptance in both the AG and NAG with no difference between the groups. However, the phases differed: In the AG, at the end of deboning, a girl looks at the breast fillets and says: ‘*yummy*, *yummy*, *yummy*’ (visually they resembled small chicken breasts), and several children also commented on how good the quail looked in the oven and how good it smelled (visually and in terms of smell, it resembled a small oven-roasted chicken). During eating, the taste was referred to as ‘like chicken’. In the NAG, familiarity was primarily displayed during the meal/tasting phase, for example, by referring to sushi, salad, and pesto in general.

Displays of liking sensory properties differed in two ways between the AG and NAG. Firstly, in the NAG, the children were focused on how different the bladderwrack felt before and after rinsing, which was not the case in the AG (rinsing of quail):

NAG: After washing the bladderwrack, a boy says: ‘*it is not that gross anymore*’ (School 3E, 43).

Secondly, in the AG, liking of taste and willingness to taste/eat were influenced by the ideational theme related to the animal dilemma/animalness. What they were eating had been an animal, and, in their mind, they had killed it:

AG: While eating, a child says: ‘*You don*’*t think about what you have done*’ (School 2D, 56).

## 4. Discussion

This paper investigated differences in the rejection–acceptance continuum mechanisms in relation to unfamiliar food items for children depending on whether or not a tactile exercise was included prior to cooking and whether or not the food was of animal origin. The applicability of the study results relates to the potential use of relevant tools in promoting children’s healthy food behavior.

### 4.1. Tactile Exercise and Food Play

With regard to the inclusion of a tactile exercise prior to cooking, our results showed that the absence of tactile exercise tended to lead to disgust-related rejection behavior. However, the inclusion of a tactile exercise to a greater extent resulted in an inappropriateness-related rejection behavior during the preparation phase (food item categorized as animal or seaweed according to the acceptance–rejection continuum). Furthermore, the tactile exercise group displayed a more playful and exploratory behavior compared to the no tactile exercise group. The playful and exploratory behavior related to the inclusion of tactile food exercises in children’s cooking classes was also found by Højer et al. [33]. Tactile food play has previously been suggested as being a successful method for promoting healthy food behavior in children. Coulthard and Sealy [36] found that, when food play was included before tasting, the number of varieties of fruit and vegetables (FV) tasted increased compared to when children did not participate in a food play session prior to tasting and when children were only visually exposed to FV prior to tasting. Nederkoorn, Theißen, Tummers, and Roefs [37] found that children who had played with jelly before eating a jelly dessert ate more than children who had only played a board game prior to eating the jelly dessert. To the knowledge of the authors, the difference between the inclusion and absence of a tactile exercise as part of a cooking session has not been investigated in previous studies. Therefore, particularly, the finding that rejection behavior seems to be driven by different mechanisms depending on the inclusion or absence of tactile play is of interest and should be investigated further in future studies aimed at promoting children’s healthy food behavior.

### 4.2. The Inappropriate (Unfamiliar) Meal: Animalness and Not Food

This study also sought to investigate whether the origin of an unfamiliar food item (animal/nonanimal) had an impact on children’s display of rejection and acceptance–related behavior throughout the cooking exercise, including tasting/eating.

Quail was chosen to represent the unfamiliar food item of animal origin since it was possible to buy it with head and feet (increased animal reference), and it is small in size, which matched the frame for preparation time. Bladderwrack was chosen as a nonanimal food item for its sensory properties: slightly slimy to the touch. Moreover, neither quail nor bladderwrack is commonly consumed in Denmark, and they were therefore deemed to be unfamiliar food items for the children.

According to Angyal [51] and Rozin and Fallon [26], disgust is closely linked to the perception of a food item being spoiled (for example, because of a slimy texture and/or inappropriate smell for that food), possessing the power to contaminate other (food) objects, or having animal-like properties. Martins and Pliner [27] found that slimy texture was a promoting factor in disgust-related rejection, but they did not find support for the animal-induced disgust perception as put forward by Rozin and Fallon [26]. In our study, disgust-related rejection behavior was particularly displayed during initial physical contact and preparation, and the contamination factor was pronounced in both the animal and nonanimal groups. In the animal group, animalness (quail waste products) was the primary reason for displaying disgust, whereas the slimy surface of the bladderwrack led to disgust in the nonanimal group.

The animal origin of food items has been found to affect perceived disgustingness by Traynor, Moreo, Cain, Burke, and Barry-Ryan [52]. They found that odor evaluation differed depending on the knowledge of the origin of the food item (animal origin was deemed to be more disgusting when knowledge of the origin was presented). In another study, Martins and Pliner [53] investigated the difference between familiar and unfamiliar foods of both animal and nonanimal origin. The results indicated that novel animal foods were considered more disgusting than novel nonanimal foods, and participants displayed less willingness to try novel foods of animal origin. In our study, we did not find a difference in willingness to taste between the animal and the nonanimal group. Most participants in both groups displayed a willingness to taste their dishes, even though tasting in the nonanimal group was characterized by slightly more caution (trying the bladderwrack pesto alone before mixing it with the pasta).

Furthermore, Martins and Pliner [53] also concluded that perceptions related to a novel food’s disgusting properties may be a predictor of people’s willingness to try it. This indicates that familiarity is a relevant factor in reducing the perception of disgust, for example, by exposing children to a broad variety of foods in terms of both origin and sensory properties. The school is an arena with the potential to do just that through, for example, cooking classes aimed at increasing food literacy and food acceptance, an approach also supported by, for example, Muzaffar, Metcalfe, and Fiese [14], Utter, Fay, and Denny [15], and Højer et al. [24].

In both the AG and NAG, inappropriateness was a driver of rejection-related behavior, but what promoted the perception of inappropriateness varied across groups. In the AG, it was promoted by a sense of feeling sorry for the quail and the animal origin parameter, whereas in the NAG, it was closely related to bladderwrack not being perceived as food. According to Rozin and Fallon [26], the perception of inappropriateness is deeply embedded in culture, but it is also related to the foodscape in which an individual moves [54]. In the AG, the feelings of sorrow for the quail and the animal theme could be closely related to the acceptance theme of personification/petification. By giving their quails names and making up small stories about their quail, the children had anthropomorphized the quail, which promoted feelings of sorrow for it when they had to debone it. This could be an expression of what Stanton [55] refers to as the Disneyfication of the animal. She argues that it is implied in Walt Disney productions (WDP) (cartoons) that animals depicted as heavily anthropomorphized should not be harmed. In addition, she underlines that the animals in WDPs are rarely depicted as dying due to the most common cause of death (slaughterhouses), and therefore WDPs are disconnected from reality. This could explain why participants in the animal group found it wrong and disgusting to ‘kill’ their quail. Furthermore, the animal dilemma was also present during eating, with the children not wanting to recall what they had just done in the deboning process (in their minds they were killing the quail). This is supported by Stewart and Cole’s [56] findings in an examination of the role of children’s movies featuring ‘talking animals’ with regard to how meat and a toy (representing a movie figure, here, ‘Babe’ the pig) are viewed by the child. Throughout Babe’s journey, the pig is subjectified, whereas the pig as meat is objectified, and the child does not connect the two. As such, animals can belong to different domains due to their perceived use by humans. Our findings also correlate with those of McGuire, Palmer, and Faber [57], who found that children (9–11 years old) will more often categorize farm animals as pets than food and find it less morally acceptable to eat meat and animal products compared to young adults and adults.

In the NAG, inappropriateness could be related to the lack of familiarity with bladderwrack as food. The most common form of seaweed eaten in Denmark is sushi wrapped in nori (dried seaweed typically made from the red algae *Pyropi yezonesis* and *P. tenera*). The two seaweeds are very different in appearance, and the nori is dried and pressed in sheets and as such, bears little resemblance to fresh seaweed. The inappropriateness, together with a perceived risk of contamination, resulted in the bladderwrack pesto being served on the side without being mixed with the pasta. In this way, the ‘safe’ familiar food (the pasta) was not contaminated with an unfamiliar food item. Since the children tasted the pesto alone, most of them concluded that it actually tasted good, and then they would mix the two ingredients. A study of children’s preferred food serving styles showed that young children (7 to 8 years old) preferred dish components to be served separately on the plate, whereas older children (12 to 14 years old) preferred a partly mixed and mixed serving style [58]. However, the study only included traditional familiar dishes. According to Fallon, Rozin, and Pliner [59], it is an expected behavior to separate foods to avoid contamination, especially if one of the food items has an anticipated distaste.

At the end of the exercise, most children across cases (AG, NAG) chose to taste and eat the food they had cooked except for the bladderwrack chips, which were highly disliked due to the taste and smell. This finding corresponds to that of Sick et al. [32], who found that children’s two main reasons for rejecting unfamiliar foods were taste and smell.

Acceptance was promoted by exploration, curiosity, and liking of taste, which corresponds to findings by Sick et al. [32] who found that curiosity and good taste were the two reasons most often given by children for acceptance of unfamiliar foods. Furthermore, acceptance was promoted when the food items were transformed into recognizable dishes, which was also found by Højer et al. [24].

Furthermore, a willingness to taste and eat the dishes could also stem from a combination of having made the food themselves and the context (school and cooking together). The effect of ‘I cooked it myself’ (pride) on the willingness to taste was also found in previous studies of children’s participation in cooking classes [12,33,60], as was the effect of context [12,33,61].

To the knowledge of the authors, no studies have tested children’s rejection and acceptance mechanisms in relation to food items of animal and nonanimal origin within the same study. In particular, our findings that inappropriateness is closely linked to how the food item is categorized (AG: animal, petification; NAG: not food), to the perceived domain depending on human use, the degree of anthropomorphization, and familiarity are interesting since they show that children perceive and react to food items of animal and nonanimal origin differently when it comes to rejection-related behavior. Nonetheless, most children chose to taste and eat their dishes despite prior rejection-related behavior. This could indicate that, even though rejection took two separate paths depending on the origin of the food item, acceptance was promoted by exploratory behavior, food transformation, ‘I cooked it myself’, and, ultimately, context.

## 5. Conclusions and practical application

### 5.1. Conclusions

With regard to the effect on the rejection–acceptance continuum mechanisms depending on the inclusion or absence of a tactile exercise prior to cooking, we found that rejection-related behavior differed. The no tactile exercise group displayed a disgust-related rejection behavior during the preparation/cooking phase, whereas the other group who did a tactile exercise displayed an inappropriateness-related rejection behavior during the preparation/cooking phase. In addition, the tactile exercise group exhibited a more playful and exploratory behavior during preparation/cooking than the no tactile exercise group.

In our investigation of the effect of the origin of the food item (animal vs. nonanimal) in relation to rejection–acceptance continuum mechanisms, we found that rejection-related behavior in both the AG and NAG was driven by disgust related to a fear of contamination, but in the NAG, the slimy tactile attribute was also a factor. However, inappropriateness was conditioned by the origin of the food item. In the AG, feeling sorry for the quail and the fact that it was an animal (they were killing the quail according to the children’s perception) were prominent factors, whereas in the NAG, the main factor was the bladderwrack not being perceived as food, probably due to lack of familiarity. In the NAG, the source of rejection was bad taste and smell with regard to the bladderwrack chips. Acceptance mechanisms present in both the AG and NAG were exploration and familiarity, though the children’s behavior differed between groups: The AG displayed a more playful exploratory behavior than the NAG, and in relation to familiarity, the AG made familiar references during deboning and eating, whereas the NAG did not do so until eating. Petification was only present in the AG. Liking generally did not differ between groups and was driven by good taste and familiarity.

### 5.2. Practical Application: Food Literacy and Health Promotion

The results from this study emphasize the importance of incorporating tactile exercises in cooking classes and food-related exercises with children in kindergarten, school, and leisure-time cooking activities since it seems to change the rejection reaction from disgust to inappropriateness. Inappropriateness could be reduced through exposure and increased familiarity.

Furthermore, we suggest that promoting children’s healthy food behavior in practice should not be concerned with only choosing food items that are deemed ‘safe’ and familiar because, despite rejection-related behavior during the cooking session, acceptance is ultimately possible due to, for example, hands-on experience and pride (I cooked it myself). Our study also shows the potential of the school as an arena for promoting children’s healthy food behavior.

## Figures and Tables

**Table 1:** Observation guide.

Categorization *	Exercise Phase	Observational Points
**Animal/seaweed**	(1) Introduction to the quail/bladderwrack.	Reactions: display of acceptance, rejection, curiosity, skills, social interaction, others.
	(2) Initial physical contact with quail/ bladderwrack.	
**Non-animal/not seaweed**	(3) Tactile exercise *(For the two no-print exercise observations*, *see point 4).*	
**Animal/seaweed-food**	(4) Food preparation. Quail: a. deboning, b. stuffing/cooking Bladderwrack: a. cutting into small pieces, b. cooking.	
**Food**	(5) The meal: tasting.	

* Categorization according to the acceptance–rejection continuum [33] with seaweed included to represent bladderwrack.

## Data Availability

The data set presented in this study are available upon request to the first author.

## Appendix A

**Table A1:** Thematic scheme across cases.

	Quail—Print (*n* = 30)	Quail—No Print (*n* = 38)	Bladderwrack—Print (*n* = 29)	Bladderwrack—No Print (*n* = 32)
**Rejection**	**Inappropriate:** Ideational: (1) feeling sorry for; (2) it is an animal	**Inappropriate:** Ideational: (1) feeling sorry for; (2) it is an animal	**Inappropriate:** Ideational—not food	**Inappropriate:** Ideational—not food
	**Distaste:** Sensory properties: touch	**Distaste:** Sensory properties: visual, touch	**Distaste:** Sensory properties: smell, touch, visual, sound, taste	**Distaste:** Sensory properties: smell, touch, visual, sound, taste
	**Disgust:** Sensory properties: touch, smell, sound, contamination, ideational—it is an animal	**Disgust:** Sensory properties: touch, smell, sound, contamination, ideational–it is an animal	**Disgust:** Sensory properties: touch, smell, contamination	**Disgust:** Sensory properties: touch, smell, contamination
**Acceptance**	**Liking:** Sensory properties: visual, taste	**Liking:** Sensory properties: visual, smell, taste, Like it, but the animal dilemma	**Liking:** Sensory properties: touch, visual, smell, taste	**Liking:** Sensory properties: touch, visual, smell, taste
	**Curiosity:** Exploration (1) in general; (2) related to play; (3) related to exploring taste	**Curiosity:** Exploration (1) in general; (2) related to play; (3) related to exploring taste	**Curiosity:** Exploration (1) in general; (2) related to play; (3) related to exploring taste	**Curiosity:** Exploration (1) in general; (2) related to play; (3) related to exploring taste
	**Personification/petification:** making up small stories	**Personification/petification:** making up small stories		
	**Familiarity:** Comparing with known food, transformation from animal to food	**Familiarity:** Comparing with known food, transformation from animal to food	**Familiarity:** Comparing with known food	**Familiarity:** Comparing with known food

**Table A2:** Meta-theme and theme essence.

Metatheme/*Theme*	Essence
**1. Rejection** *Inappropriate*, *disgust*, *and distaste*	The metatheme ‘rejection’ as a concept concerns observed behavior and dialogue reducing liking and willingness to taste or eat a food item [30]. Inappropriateness, in this case, refers to two different ideational mechanisms. One of these is based on the fact that it is not food or that it is an animal (the idea of it not being food or being an animal/that it had a life can lead to rejection due to an imagined transfer of contamination and/or cultural inappropriateness) [30,53,62]. The other is based on a respect/”feeling sorry for” paradigm [30]. Disgust refers to contamination: a fear of being soiled by either touching or being near what is perceived as (animal body) waste products [51], sensory properties: a dislike of, for example, smell, touch, and visual cues combined with elements of either inappropriateness, contamination or ideational rejection [63]. Ideation related to disgust refers to it being an animal, which promotes a strong display of emotions [30]. Distaste refers to an affective hedonic sensory-driven rejection reaction (e.g., smell, touch, taste, appearance, texture, sound) [30].
**2. Acceptance** *Curiosity*, *person/pet*, *familiarity*, *and liking*	The metatheme ‘acceptance’ as a concept concerns the promotion of a willingness to taste the food, but it can then be rejected [29]. Observed behavior and dialogue related to curiosity driven by exploration of an unfamiliar/relatively unfamiliar object. Curiosity driven by exploration is defined as a willingness to engage and investigate without fear but with a degree of uncertainty being present [64,65]. In this case, exploration relates to play (playing with the food in general), investigation, and taste (trying a novel food). Secondly, acceptance is related to the theme personification/petification driven by making up small stories about the quail/bladderwrack. Thirdly, familiarity is a theme related to comparisons with known foods and the transformation from raw material/animal to a familiar food. The fourth theme, liking, relates to positive affective hedonic sensory-driven acceptance reactions (e.g., touch, taste, appearance, smell) [29,30].
